# A robust clinical-laboratory AI model for predicting cumulative live birth per oocyte retrieval as a benchmark for evaluating emerging embryo selection technologies

**DOI:** 10.5935/1518-0557.20260057

**Published:** 2026

**Authors:** Jose G. Franco Jr, Claudia Petersen, Laura D. Vagnini, Fabiana C. Massaro, Bruna Petersen, Andreia Nicoletti, Juliana Ricci, Camila Zamara, Isabela M. Pasotti, Renata A. Pouza, Bianca C. Matuella, Elisange-la V. Espirito-Santo, Joao B. Meziara, Antonio H Oliani, Joao Batista A. Oliveira

**Affiliations:** 1 Center for Human Reproduction Prof. Franco Jr, Ribeirão Preto, Brazil; 2 Paulista Center for Diagnosis - Research and Training, Ribeirão Preto, Brazil; 3 Department of Gynecology and Obstetrics, São José do Rio Preto School of Medicine (FAMERP), São José do Rio Preto, Brazil; 4 University of Beira Interior - Faculty of Health Sciences, Covilhã, Portugal; 5 Reproductive Medicine Unit - University Hospital Centre Cova da Beira, Covilhã, Portugal

**Keywords:** IVF, ICSI, live birth rate, AI model, embryo selection, benchmark

## Abstract

**Objective:**

To evaluate the performance of a clinical-laboratory artificial intelligence (AI) model for predicting cumulative live birth rate (CLBR) per oocyte retrieval and to assess its utility as a benchmark for emerging embryo selection technologies.

**Methods:**

This retrospective cohort study included 89 completed ICSI cycles (2023-2024) from a single center. The multivariable AI model, based on hierarchical logis-tic regression, integrates female age with non-linear pe-nalization; serum AMH; number of metaphase II oocytes; fertilization rate; blastocyst formation rate and quality; presence of male factor infertility; and distinct probability coefficients for fresh and frozen embryo transfers. CLBR was calculated per oocyte retrieval, incorporating both fresh and frozen embryo transfers, representing the most clinically meaningful outcome for patients. Model discrim-ination was assessed using receiver operating character-istic (ROC) curves, with area under the curve (AUC) and 95% confidence intervals (DeLong’s method). Accuracy, sensitivity, and specificity were determined at a 50% prob-ability threshold.

**Results:**

Mean patient age was 37.2±4.3 years, AMH 2.86±2.1 ng/mL, and number of MII oocytes 7.8±3.2, fer-tilization rate averaged 79±18%, blastocyst formation rate 58±24%, mean blastocyst quality score 2.1±0.9, fresh blastocysts transferred 1.8±0.9, and cryopreserved blasto-cysts 2.1±2.0. Male factor infertility was present in 34% of cycles. The model achieved an AUC of 0.90 (95% CI 0.82-0.95). At the 50% threshold, balanced accuracy was 82%, sensitivity 82.2%, and specificity 81.8%. The confusion ma-trix revealed 7 false positives and 5 false negatives.

**Conclusion:**

This clinical-laboratory AI model provides accurate prediction of cumulative live birth per oocyte re-trieval and establishes a validated benchmark for critical-ly evaluating emerging embryo selection technologies. Its use may help ensure that technological innovations are assessed against biologically grounded outcomes before widespread clinical adoption.

## INTRODUCTION

Cumulative live birth rate (CLBR) per oocyte retrieval has emerged as the most clinically meaningful outcome in assisted reproduction. Unlike per-transfer metrics, CLBR reflects the entire reproductive potential derived from a single ovarian stimulation cycle, integrating both fresh and frozen embryo transfers originating from that cycle (Maheshwari *et al.,* 2015). For patients, this endpoint more accurately represents the probability of achieving a live birth after one complete treatment attempt and is therefore the preferred metric for counseling and clinical decision-making.

Female age remains the dominant determinant of reproductive success, yet its biological effect is markedly non-linear. The accelerated decline in oocyte competence after the mid-thirties is frequently underestimated in models that treat age linearly. Furthermore, many predictive systems incompletely integrate laboratory-derived variables that determine embryo utilization across fresh and frozen cycles. Concurrently assisted reproduction is experiencing rapid technological expansion. Artificial intelligence (AI) based embryo selection systems, time-lapse imaging, and morphokinetic algorithms are increasingly being adopted with the promise of improving embryo assessment and, consequently, pregnancy outcomes (Curchoe & Bormann, 2019). Recent studies have demonstrated AI models achieving accuracies ranging from 66.5% to 98% for embryo grading and pregnancy prediction (Nicolielo *et al*., 2025; Salih *et al*., 2025; Chaudhari *et al*., 2026), with AUC values ranging between 0.56 and 0.70 when predicting implantation or live birth (Thirumalaraju *et al*., 2026). However, these technologies are often evaluated against per-transfer outcomes or implantation rates, without consideration of cumulative success derived from a complete cycle. Moreover, the absence of a robust, biologically coherent baseline model makes it difficult to determine whether these innovations provide genuine incremental clinical benefit beyond what can be predicted using routinely available clinical and laboratory variables (Leijdekkers *et al*., 2019). Models developed on local populations are particularly important given evidence that reproductive outcomes and ovarian reserve parameters may differ across ethnic and demographic groups (Iglesias *et al*., 2014). A benchmark model calibrated to a specific population can therefore serve as a more appropriate reference for evaluating technologies intended for use in that population. The present study had two objectives: first, to develop and validate the Pardal Model, a clinical-laboratory AI algorithm predicting cumulative live birth per oocyte retrieval using variables routinely available in clinical practice; and second, to propose its performance metrics as a methodological benchmark for evaluating emerging embryo selection technologies. We hypothesize that any novel technology must demonstrate predictive superiority over this biologically grounded baseline to justify its clinical adoption and cost-effectiveness.

## MATERIAL AND METHODS

### Study Design and Population

This retrospective cohort study included 89 consecutive ICSI cycles performed between January 2023 and December 2024 at the Centre for Human Reproduction Prof. Franco Jr, Ribeirão Preto, Brazil. Cycles were eligible for inclusion if they: (1) used autologous oocytes; (2) completed all fresh and frozen embryo transfers derived from a single oocyte retrieval; and (3) had complete data for all study variables. Cycles with incomplete follow-up were excluded.

### Clinical and Laboratory Procedures

Ovarian stimulation was performed using standard protocols with recombinant or urinary gonadotrophins, with GnRH antagonist or agonist as co-treatment. Final oocyte maturation was triggered with recombinant hCG and/or GnRH agonist when at least two follicles reached ≥17 mm in diameter. Oocyte retrieval was performed 34-36 hours after trigger under ultrasound guidance. ICSI was performed on metaphase II (MII) oocytes. Fertilization was assessed 16-18 hours post-injection. Embryos were cultured in continuous single medium complete (CSCM, Irvine Scientific, USA) under oil at 37°C, 6% CO₂, and 5% O₂. Blastocyst development was assessed on days 5 and 6 post-insemination using the Gardner grading system (Gardner *et al*., 2000). Blastocyst quality was categorized as: BQ1 (low quality: Gardner grade C for inner cell mass or trophectoderm), BQ2 (intermediate quality: Gardner grade B for both parameters), or BQ3 (high quality: Gardner grade A for at least one parameter). Fresh embryo transfers were performed on day 5 or 6 under ultrasound guidance. Supernumerary blastocysts were vitrified using medium and straw (Ingamed, Brazil). Frozen-thawed embryo transfers were performed in either natural or programmed endometrial preparation cycles.

### Study Variables

The following variables were collected for each cycle: female age (years), serum AMH (ng/mL), number of metaphase II oocytes (MII), fertilization rate (FR) = (number of 2PN zygotes / number of MII oocytes injected), blastocyst formation rate (BR) = (total number of blastocysts / number of 2PN zygotes), blastocyst quality (BQ): categorical variable (1=low, 2=intermediate, 3=high), assigned as the modal quality of all blastocysts from the cycle; male factor infertility (MF): binary variable (1=present, 0=absent), defined according to WHO criteria (2021); number of fresh blastocysts transferred; number of cryopreserved blastocysts. The primary outcome was cumulative live birth per oocyte retrieval, defined as the delivery of at least one live infant after 24 weeks’ gestation resulting from any fresh or frozen embryo transfer derived from the same oocyte retrieval.

### AI Model Development

The Pardal Model (DeepSeek/AI system) was developed using a hierarchical logistic regression approach to account for the nested structure of embryos within cycles. The model first estimates the probability of implantation for each individual embryo and then aggregates these probabilities to compute the cumulative live birth probability per oocyte retrieval.

### Statistical Analysis

Descriptive statistics were calculated for all variables and presented as mean±standard deviation (SD) for continuous variables and frequencies (percentages) for categorical variables. Model discrimination was assessed using receiver operating characteristic (ROC) curve analysis. The area under the curve (AUC) was calculated with 95% confidence intervals using DeLong’s method (DeLong *et al*., 1988). At a probability threshold of 50%, a confusion matrix was constructed to visualize the distribution of correct and incorrect predictions. Performance was also stratified by predicted probability categories (0-29%, 30-49%, 50-69%, 70-89%, 90-100%) to assess model calibration across the risk spectrum. All statistical analyses were performed using R version 4.2.1 (R Foundation for Statistical Computing, Vienna, Austria).

## RESULTS

### Patient and Cycle Characteristics

A total of 89 completed ICSI cycles from 89 women were included in the analysis. The demographic and clinical characteristics of the study population were as follows: age (years) 37.2±4.3, AMH (ng/mL) 2.86±2.1, MII oocytes 7.8±3.2, fertilization rate (FR) 79±18%, blastocyst formation rate (BR) 58±24%, blastocyst quality (BQ) 2.1±0.9, fresh blastocysts transferred = 1.8±0.9, cryopreserved blastocysts = 2.1±2.0. The mean age of 37.2 years reflects a typical IVF population, with a wide range of AMH values indicating diverse ovarian reserve profiles. Male factor infertility was present in approximately one-third of cycles. The mean total of 3.9±2.3 blastocysts per retrieval provided adequate opportunities for cumulative success across fresh and frozen transfers.

### Model Performance

The Pardal Model demonstrated excellent discriminatory capacity, with an AUC of 0.90 (95% CI: 0.82-0.95). Performance metrics at the 0.50 threshold were: balanced accuracy 82%, sensitivity 82.2% (95% CI: 75.4-96.2%), and specificity 81.8% (95% CI: 67.9-92.8%). The false positive rate was 17.1% and the false negative rate 11.4%. Performance was more modest in the intermediate probability category (50-69%), where only half of predictions were correct, suggesting that this region represents a zone of clinical uncertainty where additional prognostic information may be most valuable.

## DISCUSSION

The present study describes the development and validation of the Pardal Model, a clinical-laboratory AI algorithm for predicting cumulative live birth per oocyte retrieval in ICSI cycles. With an AUC of 0.90 and balanced accuracy of 82%, the model demonstrates robust predictive performance using only variables routinely available in clinical-laboratory practice. Beyond its predictive utility, we propose that this model serves as an evidence-based benchmark against which emerging embryo selection technologies should be critically evaluated.

Several prediction models for IVF outcomes have been published, though most focus on per-transfer rather than cumulative live birth. The McLernon models, developed using the UK HFEA registry of 113,873 women, achieved indices ranging from 0.68 to 0.75 for predicting cumulative live birth over multiple complete cycles (McLernon *et al*., 2016). More recently, Xia *et al*. (2024) developed three models for CLBR prediction in 32,306 Chinese cycles, achieving indices of 0.75 (pre-treatment), 0.77 (post-stimulation), and 0.82 (post-treatment). Our model’s AUC of 0.90 compares favorably with these published models, particularly considering that our post-treatment model incorporates detailed embryological parameters, including blastocyst quality and formation rate, that may enhance discrimination.

In terms of machine learning approaches for live birth prediction, a recent comparative study evaluated multiple algorithms, including SVM, Random Forest, XGBoost, and logistic regression (Bai *et al*., 2025). The best-performing models achieved test AUCs of 0.71-0.75, substantially lower than our model’s performance. However, direct comparisons are limited by differences in outcome definition, including per-transfer versus cumulative outcomes, population characteristics, and predictor sets.

The rapid adoption of AI-based embryo selection tools necessitates rigorous evaluation against clinically meaningful benchmarks. The MAIA platform, developed for the Brazilian population, achieved an overall accuracy of 66% for clinical pregnancy prediction in prospective testing across three centers, with an AUC of 0.65 (Nicolielo *et al*., 2025). While this represents a valuable tool for embryologist decision support, its performance falls below the benchmark established by our clinical-laboratory model.

Similarly, iDAScore for aneuploidy prediction achieved an AUC of 0.61 alone, rising to 0.68 when combined with clinical characteristics (Ma *et al*., 2024), while ensemble machine learning approaches for embryo grading have reported accuracies of 93-98% for morphological classification rather than live birth prediction (Chaudhari *et al*., 2026). These comparisons illustrate a crucial point: technologies evaluated against intermediate outcomes, such as morphology, ploidy, or implantation, may not translate into equivalent improvements in cumulative live birth. Our model, by focusing on the patient-centered outcome of CLBR per retrieval, provides a more appropriate reference standard.

The principal contribution of this study lies not merely in predictive accuracy, but in establishing a benchmark against which emerging technologies can be evaluated. We propose that any novel embryo selection technology should demonstrate superiority in AUC or accuracy compared with the Pardal Model when predicting the same outcome, namely CLBR per retrieval, in the same or similar populations. It should also demonstrate incremental predictive value when added to the baseline model, quantifiable through metrics such as net reclassification improvement or integrated discrimination improvement, as well as clinical utility demonstrated through decision curve analysis showing net benefit across relevant probability thresholds.

This framework aligns with recommendations from the TRIPOD (Transparent Reporting of a multivariable prediction model for Individual Prognosis or Diagnosis) statement (Collins *et al*., 2015) and provides a structured approach to technology evaluation. Without such a benchmark, there is a risk that perceived technological gains may simply reflect favorable patient selection rather than genuine biological enhancement. The model’s incorporation of non-linear age penalization, ovarian reserve parameters, and cumulative embryo utilization captures the fundamental biological constraints on reproductive success, reducing residual confounding when evaluating downstream innovations.

Several strengths deserve mention. First, the use of cumulative live birth per retrieval as the outcome reflects the most meaningful endpoint for patients and aligns with current recommendations for reporting IVF success (Wilkinson *et al*., 2016). Second, the model explicitly accounts for the hierarchical structure of embryo data, estimating per-embryo probabilities before aggregating to cycle-level predictions. Third, the inclusion of non-linear age effects and interaction terms captures biologically plausible relationships that linear models may miss. Fourth, the model was developed using data from a single center with consistent protocols, reducing confounding from practice variation. Finally, performance was evaluated across the full probability spectrum, revealing areas of strength and uncertainty.

This study has several limitations that should be acknowledged. First, the sample size of 89 cycles, while adequate for model development, is modest and limits the precision of some estimates. External validation in larger, multicenter cohorts is essential to confirm generalizability (Steyerberg & Harrell, 2016). Second, the retrospective design may introduce selection bias, although consecutive sampling minimizes this risk.

Third, the model’s performance in the intermediate probability category of 50-69% was modest, with 50% accuracy, suggesting that this is a zone of clinical uncertainty where additional prognostic information from advanced technologies may be most valuable. Fourth, the model does not incorporate certain potentially relevant variables, such as body mass index, smoking status, or detailed sperm parameters beyond the binary classification of male factor. Fifth, temporal validation was not performed; given the rapid evolution of IVF practice, periodic recalibration may be necessary to maintain accuracy (Davis *et al*., 2017).

The Pardal Model can be implemented in clinical practice as a counseling tool, providing patients with individualized estimates of their cumulative chance of success from a single oocyte retrieval. This information can support informed decision-making about treatment continuation, embryo transfer strategies, and consideration of adjunctive technologies. For researchers and technology developers, the model provides a transparent, biologically grounded benchmark for evaluating new interventions.

Future studies of AI-based embryo selection, time-lapse systems, or metabolomic profiling should report not only the performance of the novel technology in isolation, but also its incremental value when added to a baseline model such as ours. This approach would enable rigorous assessment of whether the technology genuinely improves prediction beyond what can be achieved with routine clinical-laboratory variables.

Future research directions include external validation in independent cohorts from diverse geographic and demographic backgrounds, temporal validation to assess model stability over time, and development of an online calculator for clinical use, similar to existing tools (McLernon *et al*., 2014). Additional priorities include integration of novel biomarkers, such as AI-derived image features, into the model to quantify incremental predictive value, as well as decision curve analysis to establish clinically useful probability thresholds for treatment recommendations.

## CONCLUSION

The Pardal Model, developed using available clinical and laboratory variables from 89 ICSI cycles, demonstrates excellent predictive performance for cumulative live birth per oocyte retrieval (AUC 0.90, Accuracy 82%). Its robust discrimination establishes an evidence-based benchmark against which emerging embryo selection technologies should be critically evaluated. We propose that any novel technology whether based on time-lapse imaging, artificial intelligence morphokinetics, or metabolomic profiling must demonstrate predictive superiority over this biologically grounded baseline to justify clinical adoption and cost-effectiveness. This framework may help ensure that technological enthusiasm in assisted reproduction remains anchored to clinically meaningful improvements in patient outcomes.

P.S. - The author provides an Excel spreadsheet to calculate the probability of having at least one newborn (fresh embryos + cryopreserved embryos) obtained through a single egg retrieval at your Center, based on Pardal’s model. Request it by email: franco@crh.com.br
